# Bipolar Disorder as Comorbidity with Sjögren's Syndrome: What Can We Do?

**DOI:** 10.1155/2020/8899615

**Published:** 2020-09-11

**Authors:** Selima Chebli, Yosra Zgueb, Uta Ouali, Sana Taleb, Fethi Nacef

**Affiliations:** Psychiatry Department A, Razi Hospital, Faculty of Medicine of Tunis, El Manar University of Tunis, Tunisia

## Abstract

Neuropsychiatric manifestations in Sjögren's syndrome are common and can occur not only during its course, but also at the onset of the disease. Depression and anxiety were the most frequently described symptoms. However, the association with bipolar disorder seems to be rare and not well documented. This case report presents a patient with bipolar disorder as comorbidity with Sjögren's syndrome, suggesting that bipolar disorder could be associated with this autoimmune disease, which could lead to delaying diagnosis and treatment. A better analysis of the clinical background should be done by psychiatrists so to help early diagnosis and adapting prescription. Corticosteroids indicated in Sjögren's syndrome should be prescribed with caution in bipolar disorder.

## 1. Introduction

Bipolar disorder is a disabling chronic mental disease characterized by recurrent manic, hypomanic, and depressive episodes. Its prevalence has been estimated to be around 1 to 2%. The etiology of BD remains not entirely known and different factors have been implicated through time (genetic, biological, and psychosocial).

Recently, multiple evidences have suggested that the immune system, central nervous system, and the endocrine system are involved in the pathophysiology of BD.

In fact, the association of systemic autoimmune diseases (SADs) with BD has been reported in several epidemiological studies. Crohn's disease, autoimmune hepatitis, rheumatoid arthritis, systemic lupus erythematosus (SLE), psoriasis, and finally autoimmune thyroiditis were found to increase the relative risk of BD [1]. However, the number of published studies about Sjögren's syndrome (SS) remains limite

Sjögren's syndrome (SS) is a chronic systemic autoimmune disorder characterized by the inflammation and the lymphocytic infiltration of exocrine glands. Its hallmark symptoms are xerostomia and keratoconjunctivitis sicca [2].

In addition to the local manifestation of SS (glandular), a neuropsychiatric involvement has been described. In fact, some studies [3] have recently reported higher rates of central nervous system involvement in Sjögren's syndrome (CNS-SS) which includes psychiatric disorder. Cox and Hales [4] have suggested that the incidence of mild to moderate psychiatric and/or cognitive impairment may be as high as 80% in patients with CNS-SS. Atypical depression (characterized by irritability, agitation, and somatic symptoms), anxiety disorder, and psychotic features have been most commonly described [5].

The aim of this article is to highlight that bipolar disorder could be associated with Sjögren's syndrome and that this association has a major therapeutic impact. Moreover, we want to emphasize that psychiatric manifestations are common in patients with SS and require early diagnosis and appropriate psychotropic therapy.

Consent was obtained from the patient for publication of this paper.

## 2. Case Presentation

Mrs. G was a 54-year-old diabetic patient who was first admitted in 2013 into an internal medicine ward for brief episodes of agitation, boxing movements in the upper limbs, and pedaling movements in the lower ones. These episodes have evolved over the previous 3 years (2 to 3 times a week) and have been accompanied by occipital migraines, horizontal diplopia, and distal sensory loss.

**Figure 1:**
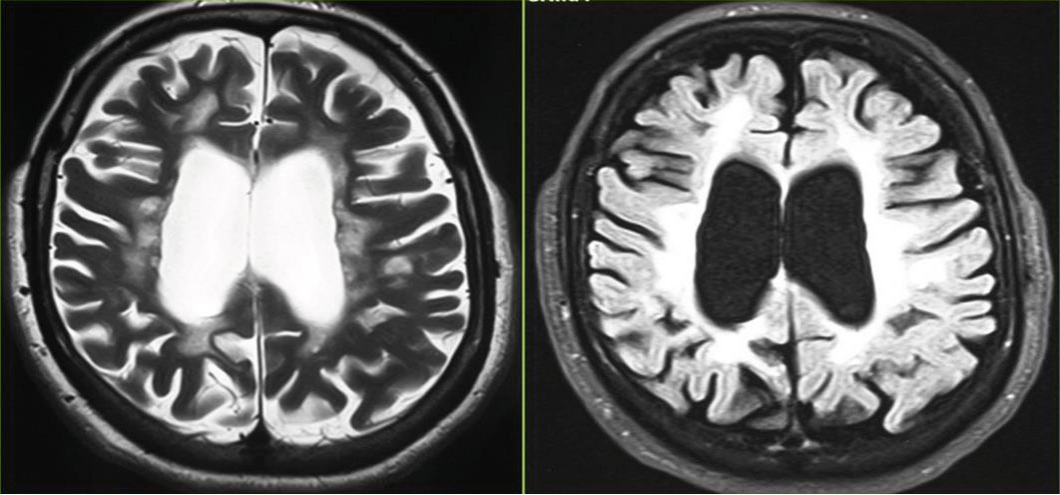
Periventricular T2/flair hypersignals associated with subcortical cortical atrophy.

Neurological examination revealed both pyramidal and posterior cordonal syndrome as well as clinical sensory abnormalities involving the left limbs and diplopia. At first, epilepsy was suspected but EEG was normal. The MRI showed periventricular white matter lesions on T2-weighted images (Figure 1). The cerebrospinal fluid analysis was normal. HIV and hepatitis C virus serologies and VDRL/TPHA were all negative. The anti-DNA test, antinuclear antibodies, and anti-SSA/Ro and anti-SSB/La tests were negative. Rheumatoid factor and total complement were normal.

Questioning the patient revealed sicca syndrome with xerophthalmia and xerostomia during the previous 6 months. A Schirmer's test was positive and salivary gland biopsies showed a lymphocytic infiltration, scored 4 using Chisholm's criteria (focus score > 1).

Mrs. G was then diagnosed as having a primary Sjögren's syndrome. Corticosteroid therapy followed by immunosuppressive treatment has been administered with no improvement. Because of persisting episodes of agitation, Mrs. G was then referred to our psychiatric ward for evaluation. At admission, she complained of irritability, agitation, difficulty in sleeping, poor concentration, and demoralization. She also reported depressed mood, anhedonia, hopelessness, feeling of guilt toward her children, and abulia.

Focused questioning revealed that Mrs. G had attempted suicide in her twenties and had suffered later from two depressive episodes with postpartum onset. An interview with Mrs. G's family revealed that she previously had an episode of antidepressant-induced mania. She also had shown periods of elevated mood, decreased need of sleep, and increase energy suggesting hypomanic episodes.

According to the Diagnostic and Statistical Manual of Mental Disorders, fifth edition (DSM5) criteria, “bipolar disorder” was diagnosed. We also diagnosed histrionic personality disorder.

A mood stabilizer was initiated (carbamazepine 600 mg/day) in association with prednisone (20 mg/day), and the patient showed a significant improvement within 4 weeks. The instability disappeared, and no more episodes of agitation or altered mood were reported.

## 3. Discussion

Psychiatric disorders associated with Sjögren's syndrome are a clinical reality. Indeed, these disorders might constitute a complication of SS due to central nervous system involvement. They can also be an early manifestation of SS, in which case they would precede the somatic symptoms [5].

According to Ampelas et al. [6], mental disorders in SS could be explained by secondary psychological distress. They have suggested that the slow progress and fluctuation course of SS created constant discomfort inducing a depressive or anxious reaction to a chronic illness. However, in line with other reports, psychiatric presentation of SS also suggests that mental disorders do not occur only as a response to a psychological distress or a reaction to a chronic disease but may be an early manifestation of the same autoimmune process, which presumes the direct immunological activity of SS on the central nervous system (by T cells, autoantibodies, cytokines, or the programmed cell death (apoptosis) [7]. It has been recently suggested that autoantibodies (autoAbs) reactive with adrenocorticotropic hormone (ACTH) and a-melanocyte-stimulating hormone (a-MSH) might be implicated in the pathogenesis of psychiatric symptomatology [8].

Several studies have reported psychiatric involvement in SS. The commonest psychiatric manifestations were depression, anxiety, and some personality traits (especially hypochondriac and histrionic features) [9]. Some investigators such as Stevenson et al. [10] and more recently, Shen et al. [3] have confirmed that depression is the most frequent comorbidity in patient with SS. However, based on the role played by autoimmunity in the etiology of bipolar disorder (BD), epidemiological studies have also reported the association of SADs with BD [11]. Wang et al. [1], in a recent study, have provided further evidence that SADs including Sjögren's syndrome are associated with higher incidence of BD.

Our case study seems to support the hypothesis that an abnormal autoimmune process is associated with increased expression of psychiatric symptoms.

Further studies on patients with Sjögren's syndrome might be needed for a better comprehension of this association as treatment and prognosis vary. Indeed, clinicians should remain vigilant for psychiatric symptoms as affective disorders are manageable if adequately treated.

Our patient showed at first no improvement under corticosteroid therapy and seemed to have more episodes of agitation. However, starting her on mood stabilizer proved to be efficient. In fact, corticosteroids, largely used in the treatment of autoimmune diseases, could induce psychiatric manifestations like manic syndrome and should be used with caution in case of association with BD [12].

Therefore, a rigorous analysis of clinical background and psychiatrist assessment should be done systematically in patient with autoimmune disease in order to avoid delays of diagnosis and to better adapt the prescription of corticosteroid/immunosuppressive agent. Appropriate additional psychiatric treatment could then lead to remarkably rapid relief as we reported in our case after initiating a mood stabilizer.

## 4. Conclusion

Neuropsychiatric manifestations in Sjögren's syndrome are common and can occur not only during its course but also at the onset of the disease. This evidence leads to important clinical considerations.

Clinicians (rheumatologists, neurologists, ophthalmologists, and psychiatrists) should be aware that psychiatric disorders are possible in SS patients (both at the onset and during the course of the autoimmune syndrome) and then may need psychiatric help and additional appropriate psychotropic therapy.

Thus, first, we suggest that the diagnosis of Sjogren's syndrome should be considered in patients who have neuropsychiatric disturbance of unexplained cause and in patients with a characteristic psychiatric profile of hysteria, somatization, and atypical mood disorder. Second, it is important to appropriately screen for mental disorders including bipolar disorder starting at disease onset, because affective disorders in SS patients are treatable and can considerably improve quality of life.
